# Therapist Attachment and the Working Alliance: The Moderating Effect of Emotional Regulation

**DOI:** 10.3389/fpsyg.2021.784010

**Published:** 2021-12-10

**Authors:** Desireé Ruiz-Aranda, Sara Cardoso-Álvarez, Javier Fenollar-Cortés

**Affiliations:** Department of Psychology, Universidad Loyola Andalucía, Seville, Spain

**Keywords:** therapist effects, attachment, working alliance, emotional regulation, therapy

## Abstract

**Objective:** To explore whether the therapist’s emotional regulation strategies moderate the relationship between therapist attachment and the working alliance from the therapist’s perspective.

**Method:** A non-experimental, descriptive correlational design was used. Sixty-three psychotherapists (6 men, 57 women) participated in this study, ranging in age from 27 to 69 years, with a mean age of 39.3 years. The therapists completed the Attachment evaluation questionnaire for adults, the Spanish Adaptation of the Working Alliance Inventory, and the Difficulties in Emotion Regulation Scale. Associations between attachment and emotional regulation traits and working alliance were examined using multilevel modeling, controlling for therapist demographics, and clinical experience.

**Results:** Moderation analyses revealed significant interaction effects between therapist attachment and emotional regulation strategies.

**Conclusion:** Attachment styles would not significantly affect the therapist’s ability to establish an adequate therapeutic alliance bond. The results show that the attachment style of the therapists interacted with their emotional regulation abilities.

## Introduction

Over time, both models and therapeutic theories have established the conditions that the patient must meet in order to achieve the goals of change and succeed in the therapeutic process. Until the 1980s, research was focused primarily on studying the final results of therapy. From this decade onwards, a growing body of research has centered on the therapeutic process, with the main focus on the processes that take place within therapy related to change (Krause and Altimir, 2016).

The contribution of the therapist to the outcome of psychotherapy has long been a topic of concern among clinicians and researchers (Lafferty et al., 1989; Castonguay and Hill, 2017). Recent research regarding the processes of change in therapy attempts to analyze the characteristics of therapists who are more effective in facilitating change compared to less effective therapists (Gimeno, 2021). One of the factors that seems to be most clearly associated with the effects of the therapist is the ability of the therapist to establish a strong working alliance with a wide range of clients (defined as the combination of agreement on goals and how to achieve the goals and the personal bond between the patient and therapist; Bordin, 1979). Recent research shows that the therapeutic relationship contributes substantially to success in all types of therapy and is important in all models and theoretical orientation (Norcross and Lambert, 2018). A recent meta-analysis indicates that the alliance explains 8% of the variance in psychotherapy outcomes, which highlights the importance of adequately considering this element (Flückiger et al., 2018).

Although studies are inconclusive regarding the relationship between therapist attachment style and the therapeutic process, previous research in psychotherapy suggests that adult therapists attachment styles are associated with the therapist’s ability to form strong therapeutic alliances with their clients (Sauer et al., 2003; Black et al., 2010; Levy and Johnson, 2019). Attachment refers to the tendency of young children to seek contact with one or more consistent caregivers when frightened, worried, or vulnerable and find such contact comforting (Bowlby, 1969; Fearon and Roisman, 2017). Bowlby had characteristically referred to the therapeutic relationship as an attachment relationship (Bowlby, 1969). Indeed, attachment theory has been regularly used as a framework for the exploration of the therapeutic relationship. A therapist can serve as a safe haven and a secure base from which clients can explore and reflect on painful memories and experiences. Securely attached therapists are more likely to form a positive therapeutic alliance, and they can create a secure base from which a client can examine meaningful experiences for change to take shape (Romano et al., 2008). They appear better able to cope with those who are distressed or who demonstrate more difficulties during therapy (Mikulincer et al., 2013). Some evidence also suggests that attachment styles and emotional abilities are correlated (Hamarta et al., 2009). Research in this field shows that people with more insecure attachment tend to have more difficulty managing anxiety in their interactions with others. However, more securely attached individuals are more trusting, open, and better handle the emotions elicited by relationships with other people (Zysberg et al., 2019). From this perspective, the ability to accept and manage emotions can aid in the effective management of interpersonal communication, relationship building, and conflict resolution, which can influence the construction and repair of the therapeutic alliance (Cann et al., 2008).

Another therapist variable associated with the establishment of the therapeutic alliance involves the emotional regulation abilities of the therapist. Previous studies indicate that a therapist’s ability to observe and examine his or her own competencies, together with his or her emotional and social abilities, correlate positively with the therapeutic alliance (Corbella and Botella, 2003). One of the essential elements of the alliance is emotional quality and the bond between therapist and patient, essential for the success of the psychotherapeutic process (Bordin, 1979). Therefore, a logical assumption is that those who are better able to manage their emotions will be able to build better relationships with their clients using the information provided by their emotions (Cherry et al., 2013). Psychotherapists with stronger emotional skills may be more effective in providing appropriate empathic responses to clients and better able to manage their own emotional responses to challenging client behaviors and emotions (Cooper and Ng, 2009; Kaplowitz et al., 2011).

Taking into account the above considerations, the objective of this study was to analyze the possible role of emotional regulation abilities as a moderator variable in the relationship between therapist attachment and the working alliance. We hypothesized that therapists with a more secure attachment style would generate a stronger therapeutic alliance. However, we expected an interaction effect, in that the beneficial effects of this type of attachment would be observed particularly in those therapists with better emotional regulation strategies.

## Materials and Methods

### Participants

The participants were 63 psychotherapists (6 men, 57 women) ranging in age from 27 to 69 years (*M* = 39.3, *SD* = 11.0), with a minimum of 5 years of experience in their profession (*M* = 11.2, *SD* = 8.62) and practicing any theoretical approach: cognitive behavioral (49.2% of the total sample), humanistic (14.3%), psychodynamic (1.59%), systemic (11.1%), or other (23.8%). All were members of the Official College of Psychologists of Western Andalusia (Spain). Descriptive information of the total sample is included in Table 1.

**Table 1:** Descriptive statistics for the total sample (*N* = 63).

	Total sample
Age	39.3 (11.0)
Gender (*n* Female/Male)	57/6
Years of professional experience *M*(*SD*)	11.2 (8.62)
Psychology perspective *n*(%total)	
Cognitive behavioral	31 (49.2%)
Humanistic	9 (14.3%)
Psychodynamic	1 (1.59%)
Systems psychology	7 (11.1%)
Miscellaneous (other)	15 (23.8%)
Attachment dimensions (CaMir-R) *M*(*SD*)	
Security	28.7 (6.00)
Family concern	13.1 (4.78)
Parental interference	8.78 (4.07)
Self-sufficiency and resentment against parents	10.9 (3.11)
Child trauma	10.9 (5.91)
Parental authority	11.5 (2.27)
Parental permission	6.97 (2.51)
Difficulties in Emotion Regulation (DERS) *M*(*SD*)	
Clarity	5.48 (1.41)
Awareness	6.06 (1.71)
Impulse/Strategies	13.8 (5.47)
Non-acceptance	11.7 (5.30)
Goals	9.44 (4.32)
Overall score	46.5 (15.0)
Working alliance (WAI-T) *M*(*SD*)	
Bond	77.5 (4.02)
Task	72.8 (6.34)
Goal	71.2 (5.91)

*CaMir-R, Short version of Attachment Evaluation Questionnaire in adults (Spanish adaptation); DERS, Difficulties in Emotion Regulation Scale (Spanish adaptation); WAI-T, Working Alliance Inventory – Therapist form (Spanish version); M, Mean; SD, Standard Deviation*.

### Procedure

Data collection was carried out using non-probabilistic snowball sampling. The participants filled out an online survey to facilitate dissemination among psychotherapists. The form included an information sheet explaining the objectives and nature of the study. The participants provided informed consent before completing the form. The study was approved by the corresponding Ethics Committee. The sample was limited to professionals with more than 5 years of professional experience in order to avoid biases arising from the lack of experience of novice psychotherapists, such as identification with the patient’s situation, insufficient emotional management in therapy in critical situations, or limited ability in a proactive and assertive attitude.

### Measures

The participants completed a sociodemographic questionnaire and three clinical scales. The scales were related to attachment, emotion regulation, and working alliance in clinical practice.

#### Attachment Evaluation Questionnaire in Adults

The short version of this instrument was used, which consists of seven dimensions and 32 items that the participant must rate on a 5-point Likert-type scale. The Attachment Evaluation Questionnaire in Adults (CaMir) measures a subject’s attachment representations based on evaluations of past and present attachment experiences and family dynamics. The dimensions of this instrument are as follows: Security (availability and support from attachment figures), Family concern and Parental interference (both associated with a concerned attachment style), Self-sufficiency and resentment against parents (associated with avoidant attachment), Child trauma (associated with disorganized attachment), and Value of parental authority and Parental permission (both refer to representations of the family structure). In this study, Cronbach’s alpha ranged from 0.44 to 0.89 (Balluerka et al., 2011).

#### Difficulties in Emotion Regulation Scale

This is a 28-item, self-report questionnaire measuring clinically relevant difficulties in emotion regulation. In the Spanish adaptation, the items are grouped into five subscales: impulse control difficulties, non-acceptance of emotional response, difficulties engaging in goal-directed behavior, lack of emotional awareness, and lack of emotional clarity. Subscales are scored on a 5-point scale ranging from 1 (never) to 5 (always). Higher scores indicate greater difficulty with emotion regulation. In our sample, Cronbach’s alpha ranged from 0.56 to 0.91 (Gratz and Roemer, 2004; Hervás and Jódar, 2008).

#### Spanish Adaptation of the Working Alliance Inventory

The version for therapists of this instrument was used to evaluate the therapeutic alliance by the therapist. This instrument comprises three dimensions and 36 items with a 7-point Likert-type scale. The dimensions of this instrument are the factors that make up the therapeutic alliance: Bond, Task, and Goal. The Spanish Adaptation of the Working Alliance Inventory (WAI-T) showed excellent reliability. In our sample, Cronbach’s alpha was 0.76 for the Bond dimension, 0.78 in Goal, and 0.82 in Task (Andrade-González and Fernández-Liria, 2016).

### Statistical Analyses

Given that both the Kolmogorov–Smirnov and Shapiro–Wilks tests were too sensitive, Z values for skewness and kurtosis were calculated to test the normality of the distribution (Kim, 2013). The cut-off of ±2.58 was established for the Z values (Mayers, 2013).

Possible correlations between psychological measures and dispositional variables (that is, age, number of years of professional experience, patients per week, and sessions per patient) were explored. Partial correlations between the psychological measures were also calculated, adjusting for the variables of age and number of years of professional experience.

**Figure 1:**
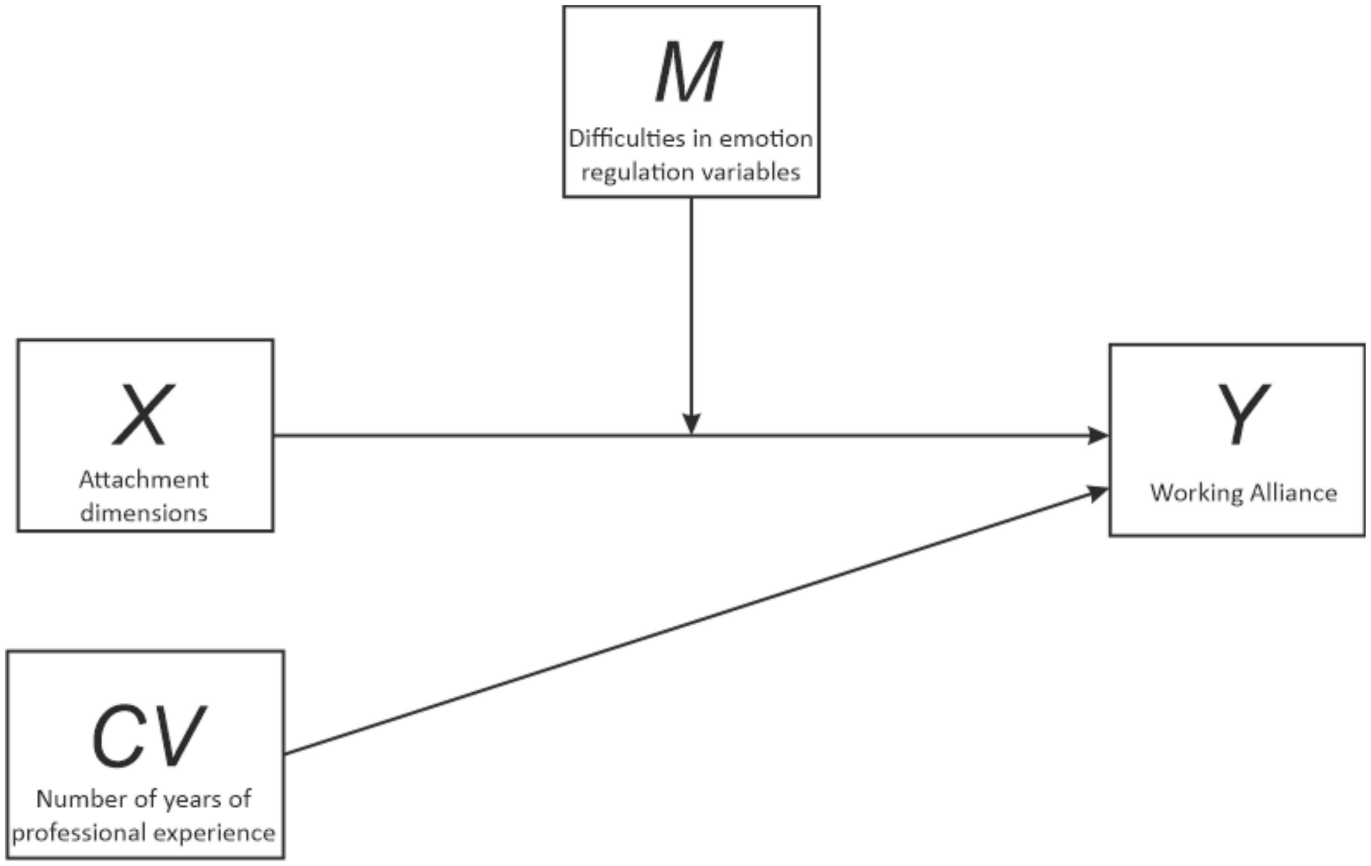
Moderation effect of difficulties in emotion regulation on the interaction between attachment dimensions and working alliance.

Finally, to examine the possible moderation effect of difficulties in emotion regulation on the interaction between the attachment and working alliance dimensions, a moderator analysis was performed with years of professional experience as a covariate using PROCESS. The outcome variables for the analysis were the working alliance dimensions. The predictor variables for the analysis were the attachment dimensions. The moderator variable evaluated for the analysis was difficulties in emotion regulation [Difficulties in Emotion Regulation Scale (DERS) total score; Figure 1].

## Results

A significant violation of the normal distribution was observed for the Difficulties in Emotion Regulation total score (Z skewness = 4.64, Z kurtosis = 4.42), Impulse Strategies (Z skewness = 5.78, Z kurtosis = 6.37), and Non-acceptance (Z skewness = 5.52, Z kurtosis = 5.90) variables. Spearman correlation analyses showed that «number of years of professional experience» was significantly correlated with Family Concern (*ρ* = −0.34, *p* = 0.006), Clarity (*ρ* = −0.26, *p* = 0.039), Non-acceptance (*ρ* = −0.26, *p* = 0.039), Goals (*ρ* = −0.26, *p* = 0.037), Bond (*ρ* = 0.31, *p* = 0.014), Task (*ρ* = 0.36, *p* = 0.004), and Goal (*ρ* = 0.31, *p* = 0.015) variables; «Age» was correlated with the Family Concern (*ρ* = −0.34, *p* = 0.006), Clarity (*ρ* = −0.36, *p* = 0.004), Non-acceptance (*ρ* = −0.29, *p* = 0.002), and Goals (*ρ* = −0.29, *p* = 0.023) variables; finally, «Sessions per Patient» was correlated with the Child Trauma (*ρ* = 0.32, *p* = 0.011), Awareness (*ρ* = −0.27, *p* = 0.031), and Task (*ρ* = −0.29, *p* = 0.022) variables.

Partial correlations between the psychological measures (adjusted for age and number of years of professional experience) showed that several of the variables were correlated (Table 2). As can be seen in Table 2, most of the significant correlations between the clinical scales were centered on the relationship between the Difficulties in Emotion Regulation and the Attachment dimension scales. Furthermore, the Difficulties in Emotion Regulation and Working Alliance scales were also highly correlated. However, significant correlations between the Attachment dimension and Working Alliance were few, and the correlations were low (from −0.26 to −0.28).

**Table 2:** Partial correlations between psychological measures (adjusted for age and number of years of professional experience; *N* = 63).

S. No.		Attachment dimensions (CaMir-R)							Difficulties in Emotion Regulation (DERS)						Working alliance	
Variable		1	2^a^^,^^c^	3	4	5^b^	6^c^	7	8^c^	9^b^	10^a^	11^a^	12^c^	13^a^	14^b^	15^b^^,^^c^
Attachment dimensions (CaMir-R)																
1.	Security	–														
2.	Family concern^c^	−0.07	–													
3.	Parental interference	**−0.67** ^***^	**0.32** ^*^	–												
4.	Self-sufficiency and resentment against parents	**−0.61** ^***^	0.09	**0.49** ^***^	–											
5.	Child trauma^b^	**−0.90** ^***^	0.21	**0.71** ^***^	**0.63** ^***^	–										
6.	Parental authority^c^	0.04	**0.30** ^*^	0.13	−0.04	0.00	–									
7.	Parental permission	**−0.48** ^***^	0.09	**0.26** ^*^	**0.46** ^***^	**0.48** ^***^	0.00	–								
Difficulties in Emotion Regulation (DERS)																
8.	Clarity^c^	**−0.31** ^*^	**0.36** ^**^	**0.44** ^***^	0.22	**0.40** ^**^	0.14	0.11	–							
9.	Awareness^b^	0.08	0.11	0.07	0.11	−0.05	0.17	−0.08	**0.35** ^**^	–						
10.	Impulse/Strategies^a^	**−0.42** ^***^	**0.31** ^*^	**0.46** ^***^	**0.34** ^**^	**0.55** ^***^	0.04	0.16	**0.56** ^***^	0.24	–					
11.	Non-acceptance^a^^,^^c^	**−0.31** ^*^	0.25	**0.38** ^**^	**0.26** ^*^	**0.48** ^**^	0.07	0.15	**0.57** ^***^	**0.27** ^*^	**0.67** ^***^	–				
12.	Goals^c^	−0.23	**0.42** ^***^	**0.29** ^*^	0.22	**0.41** ^**^	0.23	0.08	**0.51** ^***^	**0.28** ^*^	**0.60** ^***^	**−0.63** ^***^	–			
13.	Total score^a^^,^^c^	**−0.33** ^**^	**0.35** ^**^	**0.38** ^**^	**0.31** ^*^	**0.49** ^***^	0.15	0.09	**0.70** ^***^	**0.41** ^**^	**0.83** ^***^	**0.87** ^***^	**0.83** ^***^	–		
Working alliance (WAI-T)																
14.	Bond	0.15	−0.14	−0.13	−0.18	−0.24	−0.03	−0.01	−0.24	**−0.30** ^**^	**−0.41** ^**^	**−0.25** ^*^	−0.10	**−0.35** ^**^	–	
15.	Task^b^^,^^c^	0.16	−0.21	**−0.26** ^*^	−0.16	**−0.28** ^*^	−0.12	−0.08	**−0.43** ^***^	−0.28^*^	**−0.49** ^***^	**−0.42** ^***^	−0.18	**−0.43** ^***^	**0.68** ^***^	–
16.	Goal	0.19	−0.08	−0.03	−0.09	−0.25	0.00	−0.03	**−0.29** ^*^	−0.22	**−0.51** ^***^	**−0.49** ^***^	**−0.29** ^*^	**−0.50** ^***^	**0.68** ^***^	**0.81** ^***^

*CaMir-R, Short version of Attachment Evaluation Questionnaire in adults (Spanish adaptation); DERS, Difficulties in Emotion Regulation Scale (Spanish adaptation); WAI-T, Working Alliance Inventory – Therapist form (Spanish version)*.

a *Spearman’s ϱ*.

b *Number of sessions per patient also adjusted*.

c *Age adjusted*.

*The bold values are significant values:*

* *p < 0.05*;

** *p < 0.01*;

*** *p < 0.001*.

### The Effect of Difficulties in Emotion Regulation as a Moderator of the Interaction Between the Attachment Dimensions and the Bond Dimension of Working Alliance

**Figure 2:**
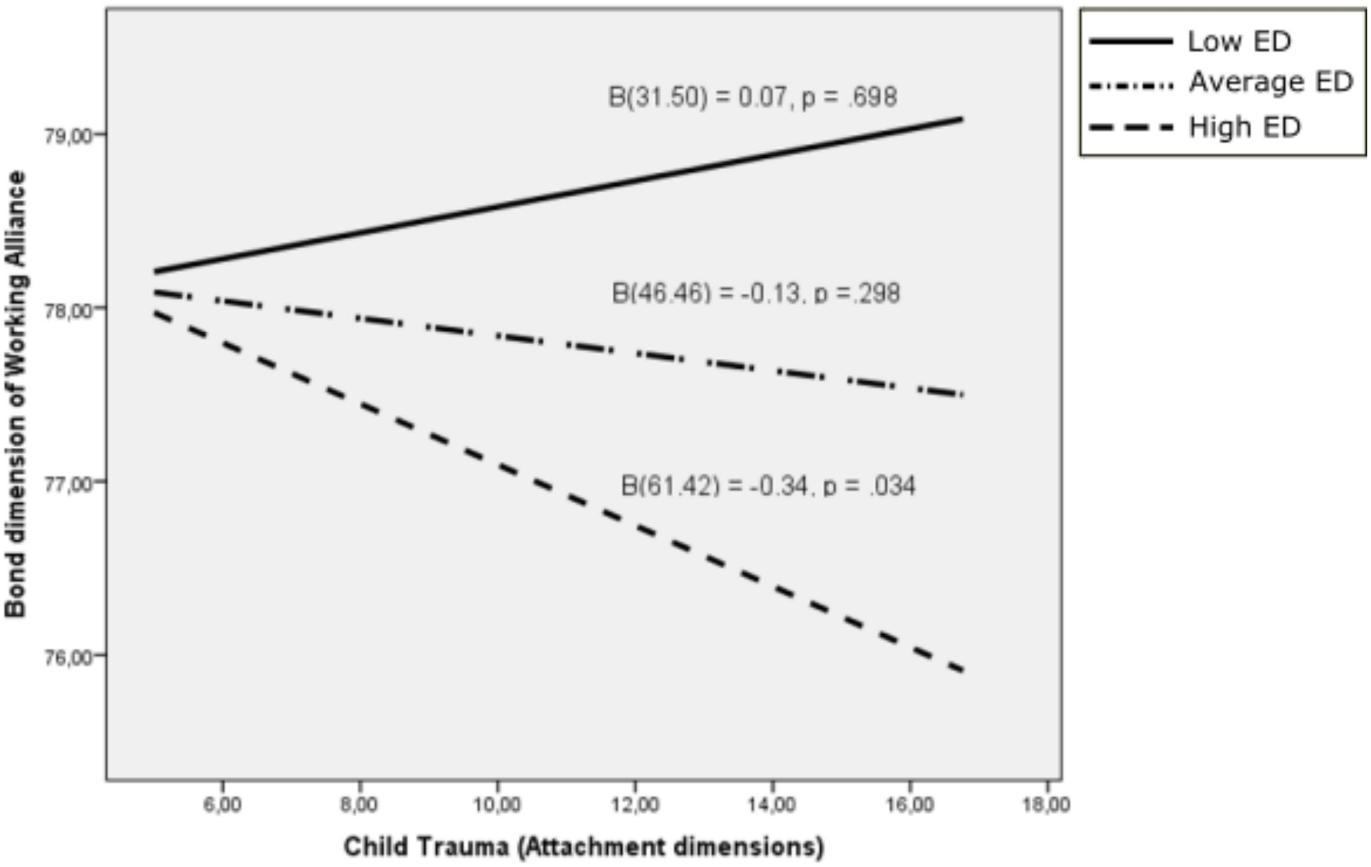
Bond dimension of working alliance as a function of child trauma: moderating effects of difficulties in emotion regulation. ED, Emotional dysregulation.

For the Bond dimension of Working Alliance, the interaction between difficulties in emotion regulation and the Security dimension of attachment was found to be statistically significant (*β* = 0.010, 95% C.I. [0.000, 0.019], *p* = 0.040). The effect of the covariate was not significant (*β* = 0.072, *p* = 198). The effect of the Security dimension of attachment on the Bond dimension of Working Alliance was statistically significant only for those with the highest scores on difficulties in emotion regulation (*ϑ*_x→y│*w* = 73.45_ = 0.290, 95% C.I. [0.000, 0.580], *p* = 0.050; Figure 2). However, the effect was not significant for those scores lower than 73.45 on the difficulties in emotion regulation (for example, *ϑ*_x→y│*w* = 73.00_ = 0.285, 95% C.I. [−0.001, 0.572], *p* = 0.051).

Although the interaction between difficulties in emotion regulation and the Trauma dimension of attachment was not significant for the Bond dimension of Working Alliance (*β* = −0.008, 95% C.I. [−0.018, 0.001], *p* = 0.116) – nor was the effect of the covariate significant (*β* = 0.080, *p* = 0.163) – the interaction was significant for the highest scores on difficulties in emotion regulation (*ϑ*_x→y│*w* = 57.00_ = −0.279, 95% C.I. [−0.558, 0.000], *p* = 0.050).

### The Effect of Difficulties in Emotion Regulation as a Moderator of the Interaction Between the Attachment Dimensions and the Task Dimension of Working Alliance

**Figure 3:**
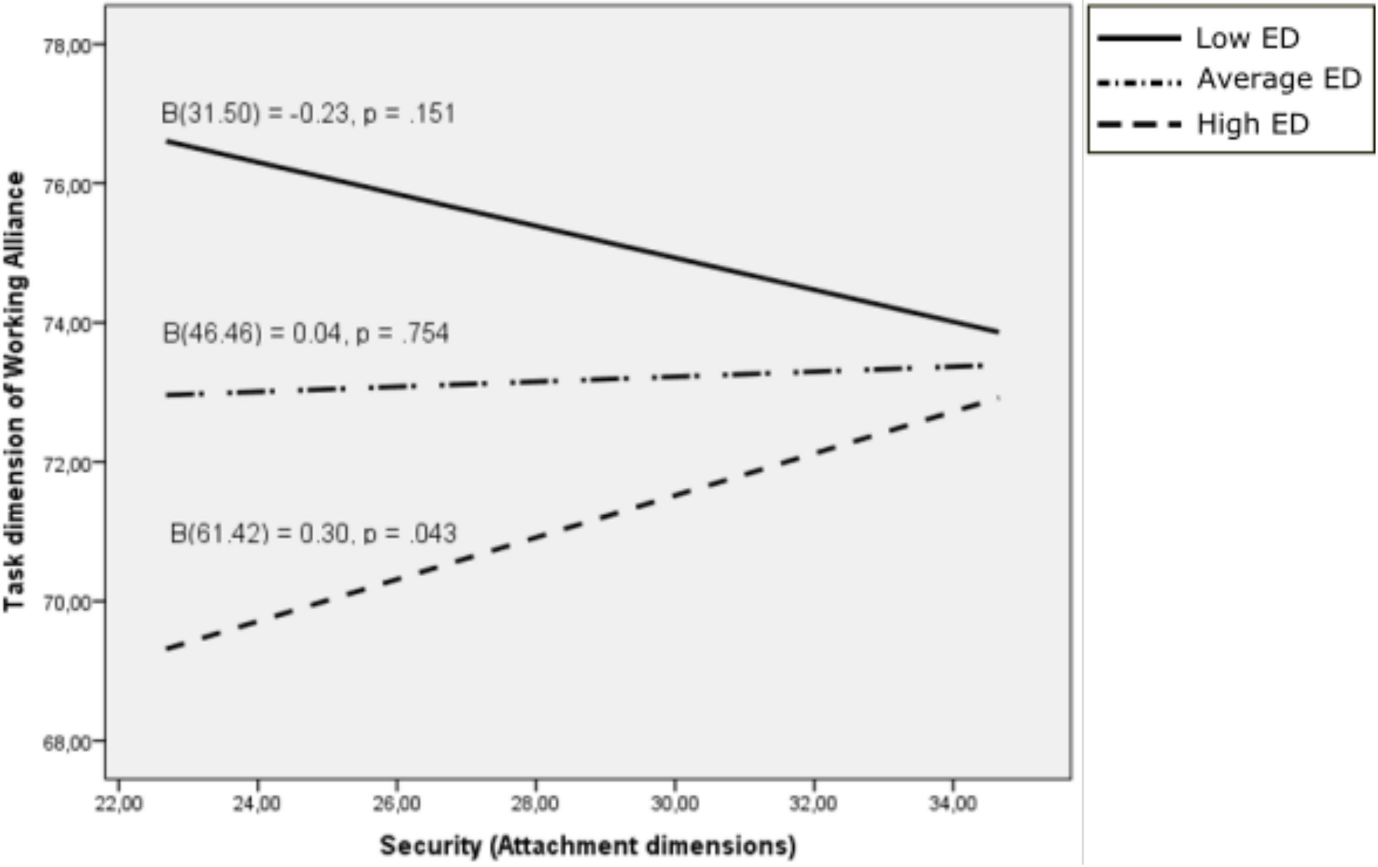
Task dimension of working alliance as a function of the security dimension of attachment: moderating effects of difficulties in emotion regulation. ED, Emotional dysregulation.

For the Task dimension of Working Alliance, the interaction between difficulties in emotion regulation and the Security dimension of attachment was significant (*β* = 0.018, 95% C.I. [0.004, 0.031], *p* = 0.010). The covariate was also significant (*β* = 0.223, 95% C.I. [0.065, 0.381], *p* = 006). The effect of the Security dimension of attachment on the Task dimension of Working Alliance was significant only for those with the highest scores on difficulties in emotion regulation (*ϑ*_x→y│*w* = 60.47_ = 0.284, 95% C.I. [0.000, 0.569], *p* = 0.0050). However, the effect was not significant for those with the middle (*ϑ*_x→y│*w* = 46.46_ = 0.036, 95% C.I. [−0.193, 0.266], *p* = 0.754) and lowest scores (*ϑ*_x→y│*w* = 31.50_ = −0.229, 95% C.I. [−0.544, 0.086], *p* = 0.151) on the Security dimension of attachment (Figure 3).

**Figure 4:**
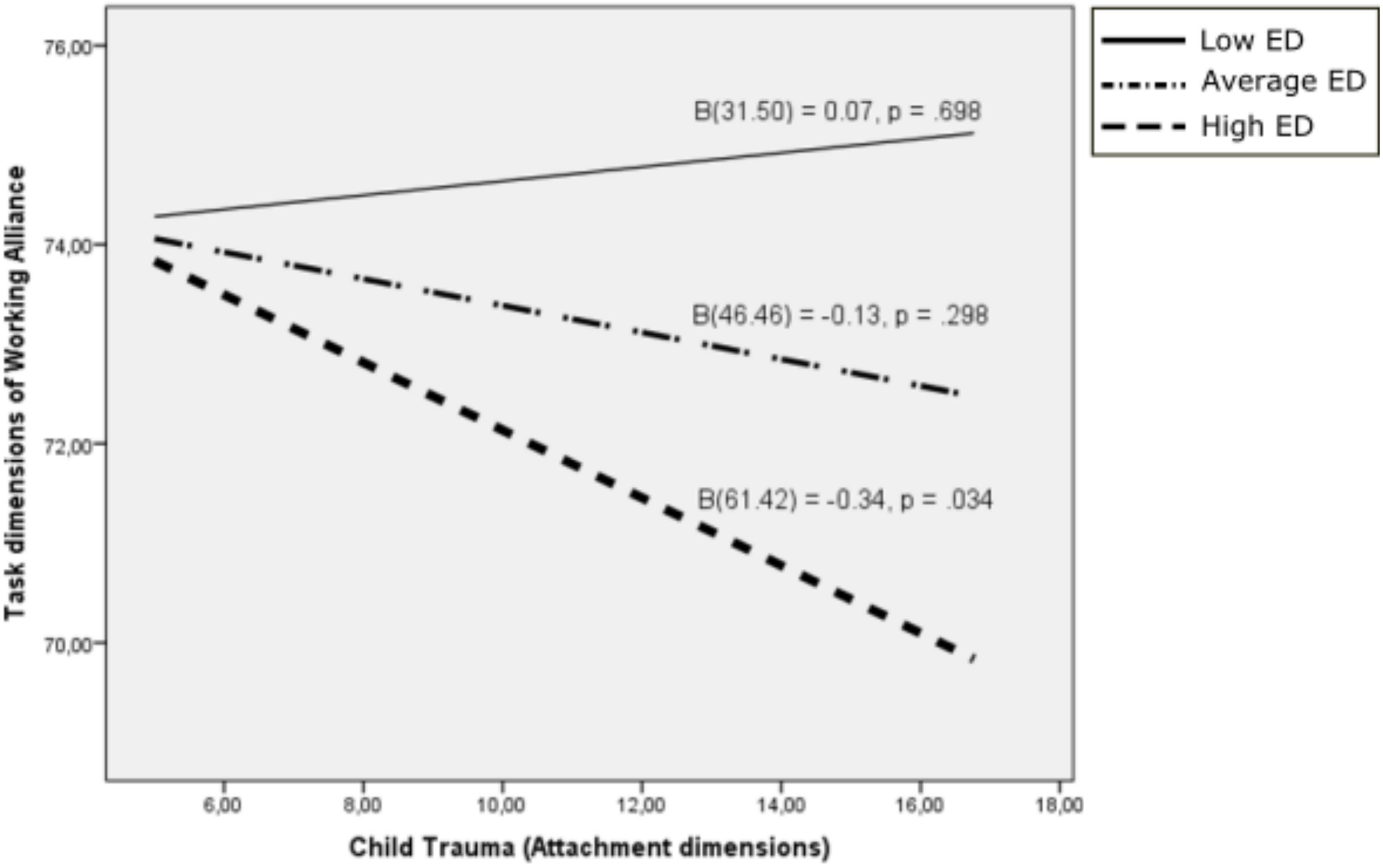
Task dimension of working alliance as a function of child trauma: moderating effects of difficulties in emotion regulation. ED, Emotional dysregulation.

Similar to the interaction between difficulties in emotion regulation and the Trauma dimension of attachment for the Bond dimension of Working Alliance detailed above, the interaction was also not significant for Task dimension of Working Alliance (*β* = −0.014, 95% C.I. [−0.029, 0.001], *p* = 0.071) – the effect of the covariate was significant (*β* = 233, 95% C.I. [−0.071, 0.395], *p* = 0.006). However, the interaction was significant for the highest scores on difficulties in emotion regulation (*ϑ*_x→y│*w* = 61.42_ = −0.340, 95% C.I. [−0.653, −0.027], *p* = 0.034; Figure 4).

### The Effect of Difficulties in Emotion Regulation as a Moderator of the Interaction Between the Attachment Dimensions and the Goal Dimension of Working Alliance

**Figure 5:**
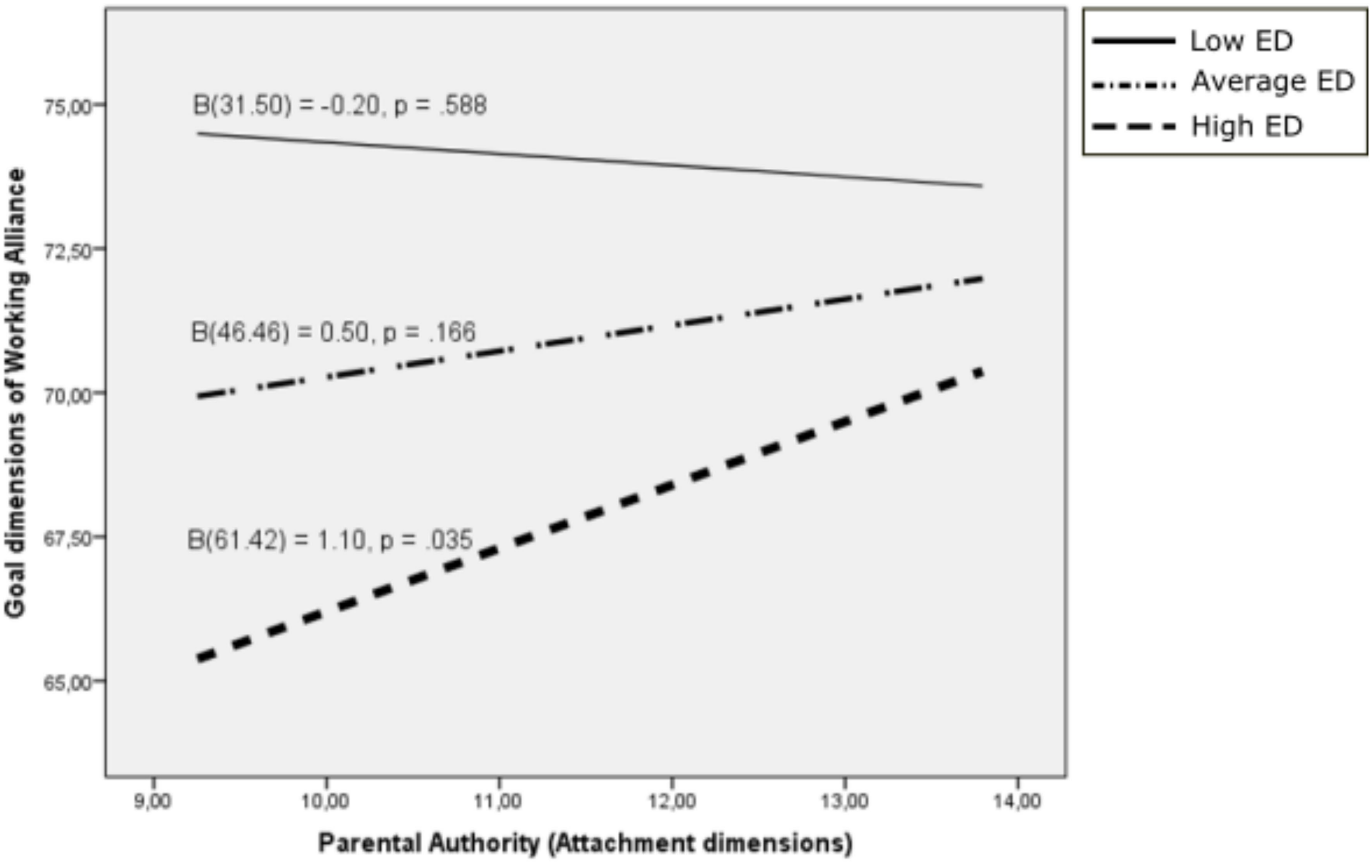
Goal dimension of working alliance as a function of parental authority: moderating effects of difficulties in emotion regulation. ED, Emotional dysregulation.

For the Goal dimension of Working Alliance, the interaction between difficulties in emotion regulation and the Parental authority dimension of attachment was also statistically significant (*β* = 0.043, 95% C.I. [0.002, 0.085], *p* = 0.039). The effect of the covariate was not significant (*β* = 0.092, *p* = 267). The effect of the Parental authority dimension of attachment on the Goal dimension of Working Alliance was statistically significant only for those with the highest scores on difficulties in emotion regulation (*ϑ*_x→y│*w* = 61.42_ = 1.099, 95% C.I. [0.078, 2.120], *p* = 0.035; Figure 5).

## Discussion

The main aim of the present study was to analyze the role of emotional regulation abilities as a moderator variable in the relationship between therapist attachment and working alliance. Overall, we found that attachment styles would not significantly affect the therapist’s ability to establish an adequate therapeutic alliance bond. As in previous studies (Ligiero and Gelso, 2002; Schauenburg et al., 2010), we found no direct influence of therapist attachment security on alliance. The results show that the therapist’s attachment interacted with his or her emotional regulation abilities. Specifically, the results indicate that having a secure attachment style is not directly related to either the Bond or Task dimension of the alliance, but that this effect is moderated by the ability of therapists to understand and manage their emotions. In line with the findings of previous studies, therapists who are able to understand and regulate their emotions are able to build a relationship of mutual trust and acceptance with clients regardless of their attachment security (Heinonen and Nissen-Lie, 2020). These skills will help foster agreement between patient and therapist regarding the tasks or activities to be performed in the therapeutic process. However, for those therapists with difficulties in accepting emotions or with limited access to effective emotional regulation strategies, it will be their attachment security that assists them in creating a quality bond with the client and agreement regarding the appropriate means to achieve the proposed goals. It seems that their perception that both in childhood and in the present, they have been sensitive to their attachment needs and have responded with affection when their protection and comfort has been needed will help to establish a quality therapeutic relationship when they lack effective emotional regulation strategies. It is possible that when securely attached therapists feel overwhelmed with their emotions, they activate the representation of how other attachment figures have behaved in their intimate relationships. A similar pattern was found for the child trauma dimension and its relationship to the bond and task dimension of the therapeutic alliance. In this case, those therapists with a representation of unavailability, violence, and threats from attachment figures during childhood will not lead to a weaker bond with the client if they have effective strategies for emotion regulation. The presence of a more insecure or preoccupied type of attachment may not affect the therapeutic alliance bond, if the professional has adequate emotional regulation strategies. As previous research has shown, the therapist’s ability to understand and manage the emotions that may be elicited by the client in the therapeutic process seems to be key in the relationship established with the client (Karson and Fox, 2010).

Similarly, the results suggest that attachment characterized by authoritative parenting would be positively associated with the goal dimension of the therapeutic alliance in those cases in which the therapist has a moderate or high level of emotional dysregulation. In this regard, it seems that the therapist’s positive evaluation of the family values of parental authority and respect for them is a characteristic of individuals with a secure attachment style. In those therapists who have difficulties in regulating emotions, this attachment style would help them to establish jointly with the client the objectives to be achieved with the psychotherapeutic intervention.

A number of limitations are also relevant to the interpretation of these findings. First, the therapeutic alliance has only been considered from the therapist’s point of view. Although both the attitudes and characteristics of the patient and those of the therapist influence the therapeutic alliance, the type of interaction they engage in will be equally important to it. The therapeutic alliance is a collaboration between patient and therapist. Thus, the constructs that both are developing concerning the work they are doing, the relationship formed, and the vision of the other are pertinent to the establishment of the therapeutic alliance (Botella and Corbella, 2011). Second, a limitation of the study is the use of self-reports to assess the skills of the therapist. As other studies suggest, in future research it would be beneficial to use measures of attachment, such as the adult attachment interview (Fleischman and Shorey, 2014) or an observer-based measure of working alliance as the Segmented Working Alliance Inventory Observer form (Berk et al., 2010; Lingiardi et al., 2011; Negri et al., 2019). In this way, the results of the research could help to deepen the role of these variables and minimize bias due to social desirability. Third, almost 50% of the therapists participating in the study used a cognitive behavioral approach. An essential characteristic, although not the only one, of good therapy of any orientation, is the strength of the alliance, which predicts success (Escudero, 2009). However, differential nuances have been raised regarding the role of the therapeutic alliance depending on the theoretical orientation of the therapy (Gunderson et al., 1997). Analyzing the influence that the therapist’s theoretical approach can have on establishing the therapeutic alliance could provide a deeper understanding of the therapeutic process.

Despite these limitations, our study has several strengths. Since most efficacy research restricts therapist variables to measures of protocol adherence (Bradley et al., 2005), few measures of therapist qualities and abilities appear in the literature. In this respect, the results of this study aids us in understanding the more or less stable characteristics of therapists that explain their differences in outcomes. In addition to contributing to further understanding of the characteristics of effective therapists, this study focused on those specific strategies that clinicians can implement to optimize the results of therapy (Norcross and Lambert, 2018; Gimeno, 2021). Improving the emotional regulation abilities of therapists will allow them to adapt to the individual characteristics of each client, since there is no single type of effective relationship. Being aware of these beneficial characteristics as therapists will allow them to enhance these abilities through deliberate practice to improve the therapeutic process (Goldberg et al., 2016).

## Data Availability Statement

The raw data supporting the conclusions of this article will be made available by the authors, without undue reservation.

## Ethics Statement

The studies involving human participants were reviewed and approved by Universidad Loyola Andalucía. The patients/participants provided their written informed consent to participate in this study.

## Author Contributions

Conceptualization: DR-A and SC-A. Methodology and formal analysis: JF-C. Writing—original draft preparation: DR-A and SC-A. Writing—review and editing: DR-A and JF-C. All authors have read and agreed to the published version of the manuscript.

## Conflict of Interest

The authors declare that the research was conducted in the absence of any commercial or financial relationships that could be construed as a potential conflict of interest.

## Publisher’s Note

All claims expressed in this article are solely those of the authors and do not necessarily represent those of their affiliated organizations, or those of the publisher, the editors and the reviewers. Any product that may be evaluated in this article, or claim that may be made by its manufacturer, is not guaranteed or endorsed by the publisher.
